# Mortality and Its Predictors among Adult Human Immune-Deficiency Virus-Infected Patients Attending Their Antiretroviral Treatment at Health Centers, Addis Ababa, Ethiopia: Multicenter Retrospective Cohort Study

**DOI:** 10.1155/2022/6128718

**Published:** 2022-09-19

**Authors:** Selam Tesfayohannes, Sisay Shine, Abinet Mekuria, Sisay Moges

**Affiliations:** ^1^Health Facility and Professionals Licensing and Control Officer at Food, Medicine, and Health Care Administrative Office, Addis Ababa, Ethiopia; ^2^Department of Health Informatics, Hosanna College of Health Sciences, Hosanna, Ethiopia; ^3^Public Health Department, Debra Berhan University, Debra Berhan, Ethiopia

## Abstract

**Introduction:**

In resource-limited settings such as Ethiopia, where the prevalence of HIV/AIDS is high, a number of factors, including economic, demographic, behavioral risk, and health factors, affect how long people with AIDS are treated with antiretroviral therapy. Since several aspects of the clinical setup may have an impact on ART patients' survival, this study was conducted in health centers. Determining the mortality rate and survival factors among adult HIV-infected patients receiving ART at health centers in Addis Abeba's Kirkos subcity is the primary goal of this study.

**Methods:**

A retrospective cohort study was carried out at the health center in Addis Abeba's Kirkos subcity. The source population consisted of all adult HIV-positive patients who were being followed up between December 1, 2014, and October 30, 2019. A total of 665 samples were collected using a computer-generated simple random sampling method in each of the three health centers that were chosen by a lottery system depending on the number of patients in the follow-up. Trained data collectors took the information out of the patient card and the electronic database. Regressions using the Kaplan‐Meier and Cox proportional hazards were employed.

**Results:**

The incidence of death rate in adult HIV-positive patients was 55 (8.5%) fatalities, translating to a death rate of 3.25 per 100 person-years. The majority of deaths occurred within 6 months of ART initiation. Predictors of mortality were: age above 50 years (AHR = 4.90, 95% CI: 2.00, 11.98), tuberculosis comorbidity (AHR = 3.46, 95% CI: 1.23, 3.33), lack of drug adherence (AHR = 1.76, 95% CI: 1.23, 3.33), co-trimoxazole therapy (AHR = 2.59, 95% CI: 1.37, 4.90), and CD4 cell count less than 200/dl (AHR = 2.77, 95% CI: 1.30, 5.92). *Conclusion and Recommendation*. Adult HIV-positive individuals had an incidence of the death rate of 55 (8.5%), which equates to 3.25 deaths per 100 person-years. Age category over 50, TB comorbidity, WHO stage IV, lack of medication adherence, co-trimoxazole therapy, body mass index under 18.5 kg/m^2^, and CD4 cell count under 200/dl were predictors of mortality. Therefore, it is important to focus on prevention, early identification, and treatment of HIV/AIDS for these predictors at all levels of the HIV/AIDS chronic care package in order to increase survival.

## 1. Introduction

The World Health Organization (WHO, 2018) reported that the human immunodeficiency virus (HIV) is one of the global and regional burdens of public health. Globally, nearly 1.7 million people are newly infected with HIV, and approximately 770,000 deaths were reported in 2018. And 32.0 million people have died from acquired immunodeficiency syndrome (AIDS)-related illnesses since the start of the epidemic [1, 2]. Because antiretroviral medication is more widely available, people with HIV/AIDS live longer and are happier (ART). Furthermore, HIV/AIDS morbidity has slightly decreased in areas where ART is widely accessible. However, developing nations like Ethiopia continue to have higher rates of mortality and morbidity [2, 3]. Although many initiatives have been implemented in Ethiopia to enhance the health and quality of life of HIV-infected patients as well as to lengthen the time between the diagnosis of HIV infection/AIDS and death, approximately 11,000 people still pass away each year as a result of HIV-related illnesses [4, 5].

The risk of mortality among patients with HIV/AIDS is approximately five times higher in patients with AIDS than in HIV-infected patients without AIDS. A viral load greater than 400 copies/mL, a CD4 count less than 200 cells/mL, TB coinfection, opportunistic infections, ART regimen, drug allergy, and low hemoglobin levels are predictors of mortality [6, 7]. However, these are not the only factors that lead to HIV/AIDS mortality [8–11]. The results of the study indicate that there are discrepancies between results from various areas of the region as well as from health centers and hospitals, both in terms of mortality rate and its predictors, which motivated this study to have a context-based investigation in this study area [12]. In addition, only a few studies were conducted in Ethiopia that looked at medical records kept in health centers; hospitals were the main focus of the majority of the research studies conducted here. As a result, in health centers, variations in the mix of clinicians, the physical setting, and the availability of research equipment can all have a distinct effect on the survival of ART patients. Thus, this study is essential for gathering information on mortality and survival indicators for HIV-infected patients receiving ART in health centers.

## 2. Methods and Materials

### 2.1. Study Areas and Settings

Addis Ababa, the capital city of Ethiopia, has 10 subcities, of which Kirkos subcity is the one located at the center of the capital. According to the 2011 estimate, the total population of the subcity was 235,441. There are a total of 7 health centers in Kirkos subcity. The health centers have departments, including ART clinics, outpatient departments (OPD), emergency departments, laboratory, pharmacy, TB, ART clinic, MCH, under five-year service, biomedical service, health information handling and management, and administration and finance department. The ART clinic is one of the departments in the health center that provides services for HIV/AIDS patients.

### 2.2. Study Design and Period

A multicenter retrospective cohort study design was used to estimate the mortality rate and its predictors among adult HIV-positive patients with ART follow-up in Kirkos subcity health centers from December 2014 to October 2019, Addis Ababa.

### 2.3. Source and Study Population

The source population consisted of all adult HIV-positive patients receiving antiretroviral therapy at health facilities located in Addis Abeba's Kirkos subcity. Study subjects selected were adult HIV-positive patients who had been receiving ART in the chosen health centers from December 1, 2014, to October 30, 2019. The study population consisted of all adult HIV-positive patients on ART in the Kirkos subcity.

### 2.4. Sample Size Determination and Sampling Procedure

The sample size was calculated by using Epi Info, Version 7 considering tuberculosis (TB) coinfection, which gives the highest sample among several factors. A hazard ratio (HR) of 2.9 was obtained, and the percentage of outcome among unexposed was 3.1% [13]. 95% was the level of confidence and 80% was the power, and the computed sample size was 577. After adding 15% of the incomplete medical records, the final sample size required for this study was calculated to be 665.

**Figure 1:**
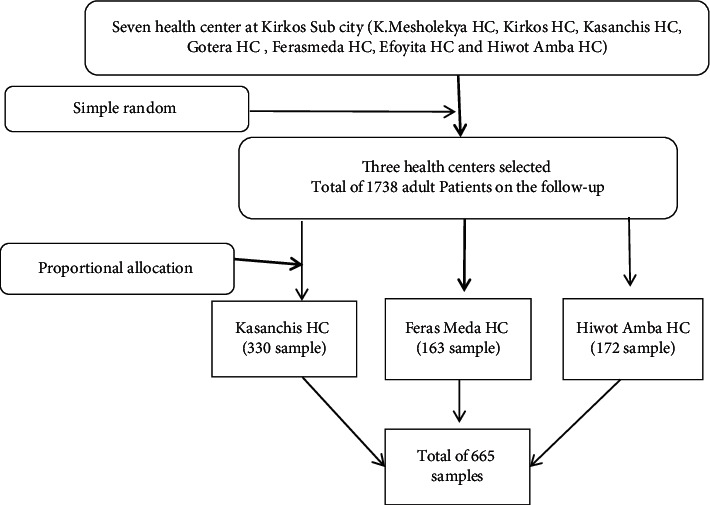
A schematic sampling procedure to study mortality and its predictors in adult human immune-deficiency virus-infected patients attending their antiretroviral treatment at health centers, Kirkos subcity, Addis Ababa, Ethiopia, 2020.

The Kirkos subcity has a total of 7 health centers; among the health centers, three (Kazanchis, Feras-Meda, and Hiwot Amba health centers) were selected randomly with the lottery method. At Kazanchis health center, there were 1225 patients, Feras-Meda had 234, and Hiwot Amba health center had 279 on follow-up. Then, study participants were selected proportionally from each of the three health centers based on the number of patients in the follow-up using a simple random sampling method. Thus, 330 samples from Kazanchis health center, 163 samples from Feras-Meda health center, and 172 samples from Hiwot Amba health center were selected using medical registration numbers (MRN) from the ART registration book. Then, a random generation of numbers was conducted using MS Excel (Figure 1).

### 2.5. Study Variables

The dependent variable was survival status (died or censored). The predictors include demographic characteristics (age, sex, educational attainment, marital status, and religion); clinical information (active TB during ART, baseline weight, WHO clinical stage, CD4 count, baseline hemoglobin, baseline BMI, and treatment); and follow-up-related information (ART regimen, drug allergy, functional status, ART regimen change, drug adherence, and co-trimoxazole therapy).

### 2.6. Operational Definition

#### 2.6.1. Death

The date of death will be recorded for all HIV-infected patients who died from all causes related to HIV/AIDS during the study period while on antiretroviral treatment.

#### 2.6.2. Censored

Patients who were alive at the end of follow-up (30th of October 2019) or lost to follow-up or transferred out were censored.

#### 2.6.3. Loss to Follow-Up

Patients missing their follow-up visits for more than 3 months, and the date of the last registered follow-up visit will be recorded as the date of loss to follow-up.

#### 2.6.4. Alive

HIV-infected patients who were still alive and using the treatment on the 30th of October 2019 will be assessed as alive.

#### 2.6.5. Survival Time

Follow-up time in a month, from the date of ART initiation to the date of death or censoring (loss to follow-up and transferred out) up to the 30th of October 2019.

### 2.7. Data Collection Methods and Tools

The antiretroviral therapy registration book and follow-up charts were the sources of data, and data were collected using a checklist developed by reviewing the national ART registration book and literature [7, 14–16], which was designed to incorporate information extracted from electronic and paper-based ART registration and follow-up charts of the ART clinic.

### 2.8. Data Processing and Analysis

Epi Data version 3.1 was used to enter data, and STATA version 14.2 was used for analysis. Descriptive statistics were used to summarize the characteristics of the cohort. The Kaplan‐Meier (KM) estimate was used to compare survival curves among the variables with categories. Both the bivariate and multivariable Cox-proportional hazard model was also employed after checking the assumption of proportionality using a global test in STATA, and both crude and adjusted hazard ratios were estimated. Those variables with *P* < 0.2 in the bivariate regression model were entered into the multivariate model. Then, variables with a statistically significant association (*P* < 0.05) in the final model were considered as independent predictors of mortality among the study participants. The result was presented using text, tables, charts, and graphs.

## 3. Result

### 3.1. Social-Demographic Characteristics of the Participants

A total of 665 HIV-infected adult patients were selected for this study, and 52 were excluded due to incomplete medical records. Thus, 613 patients were included in the study. Of these, 329 (53.7%) were female. The mean age was 38.65 (SD ± 10.32) years, and more than half of 320 (52.2%) participants' age was 35–50 years, and the majority (96.7%) were urban residents (Table 1).

### 3.2. Clinical and Follow-Up Characteristics

At the time ART was started, 488 (79.5%) of the adult HIV-infected patients were on the TDF + 3TC + EFV regimen. The cohort's mean hemoglobin at baseline was 13.41 g/dl (SD = ±3.01 g/dl). A quarter of the 143 patients (23.3%) had active TB while receiving therapy, and 97 (15.8%) of them were at WHO clinical stage IV. Over half of the 350 (57.1%) people surveyed had no co-trimoxazole medication (Table 2).

### 3.3. Mortality and Survival Analysis

**Figure 2:**
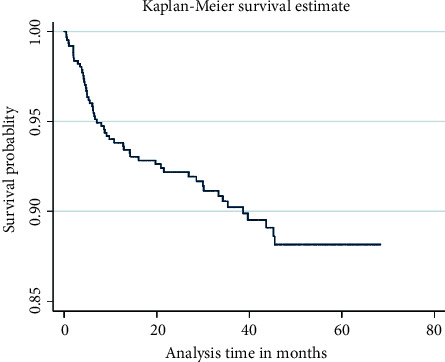
Kaplan‐Meier survival estimate in adult HIV-infected patients under ART follow-up in health centers at Kirkos subcity Addis Ababa, Ethiopia, 2014–2019.

The cohort contributed 1,693 person-years of follow-up with a median follow-up of 34 months overall (interquartile range, 33 months). The incidence of the death rate among the 613 adult HIV-infected patients under follow-up was 55 (8.5%), yielding a death rate of 3.25 per 100 person-years; 51 (8.3%) of them were lost to follow-up; 44 (7.2%) of them were transferred to different ART sites; and 463 (88.6%) were alive by December 31st, 2019. Eleven (20%) of the 55 patients who passed away did so within three months of the start of their treatment. The average time of survival was 62.35 months, with a 95 percent confidence interval (60.87 and 63.84). The overall expected survival time following the start of ART was 68.27 months, according to the Kaplan‐Meier survival calculation (Figure 2).

### 3.4. Predictors of HIV Mortality among Adult Patients

In the bivariable Cox proportional regression analysis, age of participants, occupation, marital status of widowed and divorced, active TB status, WHO clinical stage, functional status, Co-trimoxazole therapy, BMI, CD4+ counts, and baseline hemoglobin level <10 were considered as potential predictors and were included in the multivariable Cox proportional model.

The test of proportional hazards assumption based on Schoenfeld residual (Phtest), also known as the Cox proportional model assumption, was verified using a global test in the multivariable model. All variables with *P*values ≤0.2 were then exported to a multivariable Cox proportional hazard regression model after running a bivariable for each predictor. Age, active TB, WHO clinical staging, baseline functional status, co-trimoxazole medication, baseline BMI, and baseline CD4+ count were independent predictors of death in the adjusted Cox regression model in the multivariable Cox proportional hazard regression analysis.

Adult HIV patients with an age category above 50 years have a 4.89 times higher hazard of death compared to those aged 17–34 years (AHR = 4.90, 95% CI: 2.00, 11.98). Patients with TB comorbidities were highly associated with an increased risk of mortality; thus, the hazard of death was 3.46 times higher than those without active tuberculosis (AHR = 3.46, 95% CI: 1.52, 7.91). Patients at WHO stage IV were at an increased risk of death compared to their counterparts (stage I) (AHR = 4.55, 95% CI: 1.72, 12.02). Regarding functional status, bedridden patients were 2.6 times higher than the risk for ambulatory patients (AHR = 2.89, 95%CI: 1.26, 6.60). Drug adherence was also significantly associated with mortality. Thus, patients who were reported with a lack of drug adherence had a greater risk of death (AHR = 1.76, 95% CI: 1.23, 3.33). Taking co-trimoxazole therapy decreased the risk of death by 61%. There was 2.56 times the risk of death for patients not taking co-trimoxazole therapy (AHR = 2.56, 95% CI: 1.25, 5.24). Patients with a BMI of less than 18.5 had a greater hazard of death compared to its counterpart (AHR = 2.59, 95% CI: 1.37, 4.90), and patients with a CD4 cell count below 200/ul had a 2.7-fold higher risk of mortality (AHR = 22.77, 95% CI: 1.30, 5.92) than individuals with a CD4 count ≥200/ul after controlling other factors (Table 3).

## 4. Discussion

The mortality of patients enrolled in ART and risk variables for patient mortality under ART follow-up were evaluated in this study. A death rate of 55 (8.5%) was recorded out of the 613 adult HIV-infected patients under follow-up, or 3.25 deaths per 100 person-years. A study conducted at Aksum hospital in northern Ethiopia revealed a similar result, with an overall death rate of 3.2 per 100 person-years [14]. Lower than studies done in Addis Ababa, Ethiopia, where the mortality incidence was 3.8/100 person-years, 3.9 per 100-month person year in Harar, Ethiopia, and another higher mortality rate of 10.3%; 5.4 deaths/100 person-years was reported from a study conducted at seven universities based on national ART follow-up data; and 3.4 deaths per 100 person-years in Dilla. [8, 10, 15, 17], and the present study finding is higher than the study findings done in southern Ethiopia, 2.03 per 100 person-years [7], eastern Ethiopia 1.89 deaths per 100 person-years [18], and the study was done in Uganda [19]. The reason for such differences could be due to differences in study settings or differences due to the difference in the quality of care and service provision.

According to the current study, the majority of deaths (46%) happened within 6 months of their follow-up, with 11 (20%) patients dying within the first 3 months. A consistent finding was found in a hospital-based retrospective study in western Ethiopia, in which the study found that the majority of deaths (60%) occurred within the first six months [18]. Another multisite prospective cohort research conducted in Ethiopia found that 70% of deaths occurred within six months of initiating ART [10]. The explanation for this might be that HIV patients typically present to the hospital at an advanced clinical stage, and a lack of treatment adherence at the early stage of ART initiation could lead to increased mortality. As a result, test and treatment strategies that allow for quick treatment initiation regardless of the clinical condition of the clients should be enhanced.

In the present study, patients who are above 50 years have a 4.89 times higher hazard of death compared to patients in the age category of 17–34 years. A consistent finding was reported from previous studies conducted in Uganda [19] and western Ethiopia [18]. The reason could be the fact that old age is associated with immunologic suppression, exposure to infectious diseases, psychosocial comorbidities, and other factors of disease progression.

In the present study, it was demonstrated that patients with TB comorbidity were highly associated with an increased risk of mortality, and the hazard of death among patients with TB comorbidity was 3.46 times higher than without active tuberculosis. A consistent finding was reported from another similar investigation from Ethiopia, and the risk of death for patients who lived with tuberculosis was about 2.8-fold times higher than those patients who were negative and the same was also reported from a study conducted at two hospitals and six health centers in Illubabor and Buno Bedele zone [16]. Moreover, a report from Uganda showed that mortality from TB coinfection was 1.81 times higher [19]. And based on a research report from eastern Ethiopia, TB coinfection at baseline or later was also associated with an increased risk of mortality [20]. A similar finding was reported in other studies [13, 21]. The explanation for this may be that TB coinfection reduces the patient's immunity.

Advanced WHO clinical stages (stage IV) have been consistently reported as risk factors for mortality in several studies from Ethiopia [10, 17, 20, 22]. The current study found a similar result, indicating that patients with WHO stage IV were at higher risk of mortality than their counterparts (stage I), with the risk of death being 4.1 times higher. Mortality among patients with AIDS was nearly halved in the HAART era but remains approximately 5 times higher in patients with AIDS than in HIV-infected patients without AIDS [6]. And this study demonstrated that drug adherence is a significant predictor of mortality. A study conducted in western Ethiopia revealed similar findings in which nonadherent participants had a mortality of 42.5 deaths per 100 person-years and were two times as likely to die as adherent participants [18, 19]. Some of the similar previous cohort studies demonstrated that a higher CD4 cell count would reduce morbidity and mortality [20–22]. We demonstrated that patients whose CD4 cell count was <200 cells/mm3 had a higher risk of death compared to patients with a CD4 cell count ≥ of 500 cells/mm3. The study showed that the CD4 cell count was an independent predictor of AIDS progression, and it is a consistent finding with other research results conducted outside of Ethiopia [9, 23] which indicated that AIDS progression to death was clustered among patients starting therapy with a lower CD4 cell count. Thus, emphasizing the need for early diagnosis, linking, and engaging patients in a comprehensive ART care program could help reduce mortality rates.

The current study also found that baseline BMI was an important independent predictor of death. Patients with BMI <18.5 kg/m^2^ had a greater hazard of death as compared to those whose BMI was ≥18.5 kg/m^2^ and a consistent finding was reported from a study in Somalia. Thus, the risk of death in patients with a BMI <18.5 kg/m2 was more than two times higher than those whose BMI was ≥18.5 kg/m^2.20^. Furthermore, another study conducted in Cameroon showed that patients whose BMI were <15 kg/m^2^ had a three times higher risk of death than those whose BMI was >18.5 kg/m. [24] This could be due to malnutrition, suppressing immunity, the aggregate effects of malnutrition-induced immune system dysfunction, a higher burden of opportunistic infections, metabolic derangement, and anthropometric variations [25].

In the present study, adult HIV-infected patients who were bedridden at ART initiation had a higher risk of mortality compared to patients with working functional status at treatment initiation. The finding is consistent with a study conducted in eastern Ethiopia [20], southern Ethiopia [15], and those described elsewhere [22, 26, 27]. However, it was not reported as a significant predictor in a study conducted in western Ethiopia [18]. It infers the clinical truth of being bedridden is the result of suffering from advanced opportunistic infection, leading to death if there is no early initiation of the HIV/AIDS care package.

Co-trimoxazole is a feasible, well-tolerated, and inexpensive intervention for people living with HIV to reduce HIV-related morbidity and mortality [28]. However, the current study demonstrated that not taking co-trimoxazole prophylaxis was significantly associated with mortality. Thus, patients who had not taken co-trimoxazole prophylaxis have a 2.56 times higher risk of death compared to its counterpart. A consistent result was demonstrated by a study conducted in Harari, Ethiopia. Thus, not taking co-trimoxazole prophylaxis treatment (CPT) at baseline has a higher risk of death [17]. The finding was also in line with the study conducted in Uganda [19] and other parts of Ethiopia [7]. This finding implies that co-trimoxazole is a lifesaving medication in the fight against opportunistic infections that increase the incidence of mortality.

As it requires an evidence-based approach to improve survival and to manage predictors of HIV-positive patients, intervening on each predictor will generally support the achievement of agreed-upon targets and policies, such as ending the HIV/AIDS epidemic in 2030, reducing deaths by 80% by 2020, and addressing opportunistic infections such as tuberculosis as well as comorbid conditions. The study also supports Ethiopia's National Strategic Plan for HIV 2021–2025 (NSP) objective of achieving national HIV epidemic control by 2025 by lowering the rate of new HIV infections and AIDS-related deaths to under 1 per 10,000 people. The study's conclusions concur with the six strategic goals, which state to improve the creation and use of strategic information more quickly [29].

### 4.1. Limitations of the Study

Due to the retrospective nature of the data, selection bias may have been created since patients with incomplete records of variables were omitted. This is true even if the amount of inaccurate and missing data was reduced by double-checking patient medical records with digital databases. The mortality results for such patients may also be exaggerated because it is impossible to determine the status of those who were lost to follow-up.

## 5. Conclusion and Recommendation

In the 613 adult HIV-infected patients under follow-up, the incidence of the death rate was 55 (8.5%) , translating to a death rate of 3.25 per 100 person-years; among them, 51 (8.3%) were lost to follow-up; and 44 (7.2%) of them were moved to different ART sites. There were 62.35 months on average that people survived. The Kaplan‐Meier survival calculation gave a total anticipated survival time of 68.27 months after the start of ART. With a frequency of 3.25 deaths per 100 person-years, the first six months after the start of ART were discovered to be the period of greatest mortality. Age category over 50, TB comorbidity, WHO stage IV, lack of treatment adherence, co-trimoxazole therapy, BMI less than 18.5 kg/m2, and CD4 cell count less than 200/dl were all indicators of mortality. Therefore, in order to maximize CD4 count and improve survival, the HIV/AIDS chronic care package must place a strong emphasis on these predictors for early screening, prevention, early detection, and treatment of such predictors.

## Figures and Tables

**Table 1:** Sociodemographic characteristics of patients under ART in health centers at Kirkos subcity Addis Ababa, Ethiopia, 2014–2019.

Variables	Frequency	Percentage
Sex		
Male	284	46.3
Female	329	53.7

Age category (years) mean (±SD)	38.65 (±10.32)	
17–34	219	35.7
35–50	320	52.2
>50	74	12.1

Residence		
Urban	593	96.7
Rural	16	2.6

Educational level		
No Education	166	27.5
Primary education	195	32.3
Secondary education	187	31.0
Tertiary education	56	9.3

Occupation		
Formal employee (^*∗*^Gov't/^*∗*^NGO)	177	29.1
Daily laborer	320	52.6
Farmer/housewife	111	18.3
Formal employee (Gov't/NGO)	177	29.1

Marital status		
Single	310	50.6
Married	158	25.8
Widowed	55	9.0
Divorced	90	14.7

Religion		
Protestant	92	15.0
Orthodox	382	62.3
Muslim	117	19.1
Others (Catholic, Adventists)	17	2.8

*Note.* Gov't: government, NGO: nongovernmental organization, others: catholic and Adventists.

**Table 2:** Clinical characteristics of patients under ART in health centers at Kirkos subcity Addis Ababa, Ethiopia, 2014–2019.

Variables	Frequency	Percentage
Baseline hemoglobin (g/dl) mean (±SD)	13.25 (±3.16)	
<10	33	5.4
≥10	577	94.6

Active TB during treatment		
No	470	76.7
Yes	143	23.3

WHO clinical stages		
Stage I	277	45.2
Stage II	145	23.7
Stage III	94	15.3
Stage IV	97	15.8

Base line CD4+ (cells/*μ*l) mean (±SD) (*n* = 613)	391.2 (±143.5)	
<200	67	10.9
≥200	546	89.1

Functional status		
Working	401	65.4
Ambulatory	168	27.4
Bedridden	44	7.2

Drug allergy		
No	376	61.3
Yes	237	38.7

Drug adherence		
No	157	25.6
Yes	456	74.4

Body mass index		
<18.5	183	29.9
≥18.5	428	69.8

Baseline ART regimen		
ABC + 3TC + EFV	96	15.7
TDF-3TC-EFV	488	79.6
Others	29	4.7

Change in the ART regimen from baseline		
No	531	86.6
Yes	82	13.4

Co-trimoxazole therapy		
No	350	57.1
Yes	262	42.7

*Note.* ART-antiretroviral therapy, ABC-abacavir, 3TC-lamivudine, EFV-efavirenz, and TDF-tenofovir disoproxil fumarate.

**Table 3:** Bivariable and multivariable Cox-proportional regression analysis on predictors of mortality in adult patients under ART in health centers at Addis Ababa, Ethiopia, 2014–2019.

Variable	Survival status		CHR (95% CI)	AHR (95% CI)
	Censored	Died		
Age				
17–34	210	9	1	
35–50	298	22	1.67 (0.76, 3.62)	2.07 (0.90, 4.79)
>50	49	25	10.48 (4.88, 22.55)^*∗∗*^	4.89 (2.00, 11.98)^*∗∗*^

Occupation				
Formal employee (Gov't/NGO)	168	9	1	
daily Laborer	292	28	1.69 (0.80, 3.59)	1.72 (0.74, 3.98)
Farmer/housewife	93	18	3.47 (1.56, 7.74)^*∗*^	1.41 (0.55, 3.64)

Marital status				
Single	293	17	1	
Married	145	13	1.63 (0.7953.37)	1.75 (0.73, 4.17)
Widowed	44	11	3.96 (1.85, 8.477)^*∗∗*^	0.92 (0.31, 2.72)
Divorced	76	14	3.06 (1.51, 6.21)^*∗∗*^	1.79 (0.75, 4.24)

Active TB status				
No	455	15	1	1
Yes	103	40	8.54 (4.78, 15.25)^*∗∗*^	3.46 (1.52, 7.91)^*∗∗*^

WHO clinical staging				
stage I	271	6	1	1
stage II	140	5	1.27 (0.40, 4.01)	0.43 (0.11, 1.59)
stage III	79	15	6.73 (2.74, 16.52)^*∗∗*^	1.78 (0.57, 5.56)
Stage IV	68	29	14.64 (6.40, 33.49)^*∗∗*^	4.55 (1.72, 12.02)^*∗∗*^

Functional status				
Working	381	20	1	1
Ambulatory	155	13	1.40 (0.70, 2.81)	0.96 (0.423, 2.197
Bedridden	22	22	11.56 (6.33, 21.11)^*∗∗*^	2.89 (1.26, 6.60)^*∗*^

Drug allergy				
No	355	21	1	1
Yes	203	34	2.50 (0.46, 4.28)	0.95 (0.46, 1.95)

Drug adherence				
Yes	425	31	1	1
No	133	24	2.16 (1.27, 3.67)	1.76 (1.23, 3.33)^*∗*^

Co-trimoxazole therapy				
No	308	42	2.30 (1.25, 4.22)^*∗*^	2.56 (1.25, 5.24)^*∗*^
Yes	248	14	1	1

BMI				
<18.5	151	32	3.11 (1.83, 5.28)^*∗*^	2.59 (1.37, 4.90)^*∗∗*^
≥18.5	405	23	1	

CD4^+^ counts				
<200/ul	39	28	9.012 (5.33, 15.22)^*∗*^	2.77 (1.30, 5.92)^*∗*^
≥200/ul	518	28	1	

Baseline hemoglobin				
<10	15	18	12.09 (6.86, 21.31)^*∗*^	0.612 (0.28, 1.33)
≥10	540	37	1	1

*Note.* ^*∗*^ *P*=0.01 − 0.05, ^*∗∗*^*P* < 0.01, COR: crude odds ratio; AOR = adjusted odds ratio; and CI = confidence interval.

## Data Availability

The datasets used and/or analysed during the current study are available from the corresponding author or principal investigator on reasonable request.

## Abbreviations

AIDS: Acquired immune deficiency syndrome
AOR: Adjusted odds ratio
ART: Antiretroviral therapy
CD4: The cluster of differentiation 4
COR: Crude odds ratio
HIV: Human immunodeficiency virus.
